# Rapid Screening of Lipase Inhibitors from Ophiopogonis Radix Using High-Performance Thin Layer Chromatography by Two Step Gradient Elution Combined with Bioautographic Method

**DOI:** 10.3390/molecules27041155

**Published:** 2022-02-09

**Authors:** Xue Hua, Hui-Jie Hong, Dai-Yan Zhang, Qiao Liu, Fong Leong, Qi Yang, Yuan-Jia Hu, Xiao-Jia Chen

**Affiliations:** 1 Institute of Chinese Medical Sciences, State Key Laboratory of Quality Research in Chinese Medicine, University of Macau, Macao 999078, China; mb75819@connect.um.edu.mo (X.H.); yc17534@um.edu.mo (H.-J.H.); yc07528@um.edu.mo (D.-Y.Z.); yb87502@um.edu.mo (Q.L.); yb37501@um.edu.mo (F.L.); mc05849@um.edu.mo (Q.Y.); 2 Zhuhai UM Science & Technology Research Institute, Zhuhai 519031, China

**Keywords:** Ophiopogonis Radix, high-performance thin layer chromatography, two step gradient elution, bioautography, lipase inhibitors

## Abstract

In this study, a high-performance thin layer chromatography (HPTLC) method by two step gradient elution with two mobile phases was developed for the simultaneous analysis of seven constituents in Ophiopogonis Radix. The chromatography was performed on silica gel 60 F_254_ plate with dichloromethane-methanol-ethyl acetate-water (70:25:12:3, *v*/*v*/*v*/*v*) and dichloromethane-methanol (300:1, *v*/*v*) as the mobile phase for two step gradient elution. Then, the HPTLC profiles were observed after derivatization with 10% sulfuric acid in ethanol solution. The obtained HPTLC images were further analyzed by chemometric approaches and the samples could be clustered based on regions and/or growth years, which were two important factors affecting the constituents in Ophiopogonis Radix. Furthermore, five compounds including ophiopogonin D, ophiopojaponin C, ophiopogonin D’, ophiopogonin C’ and methylophiopogonanone B were screened as potential lipase inhibitors from Ophiopogonis Radix by the HPTLC-bioautographic method. The binding modes and interactions between the five compounds and lipase were further explored by molecular docking analysis. The developed HPTLC method could be used for quality control of Ophiopogonis Radix and screening of the potential lipase inhibitors.

## 1. Introduction

Obesity, which means abnormal or excessive fat accumulation, is considered as one of the greatest threats to human health. According to the World Health Organization, more than 650 million adults (18 years and older) and over 340 million children and adolescents aged 5–19 were overweight or obese in 2016 [1]. In Asia especially in China, obesity has surged insurmountably from 2007 to 2016 as an increasing rate at 3.7–6.6% [2]. Obesity tends to aggravate the chances of acquiring diseases such as type 2 diabetes, hypertension, fatty liver, and cancer, which in turn reduces both life expectancy and quality of life.

Pancreatic lipase is a key enzyme for the digestion of triacylglycerols, and inhibition of lipase has been the most explored strategy for the treatment of obesity until now. However, orlistat, the only lipase inhibitor approved clinically, has several adverse effects such as severe liver and kidney injury [3,4]. In recent years, more and more medicinal herbs have been reported to show inhibitory activities against pancreatic lipase, such as *Morus alba* [5], *Atractylodes lancea* [6], *Forsythia suspensa* [7], and *Camellia nitidissima* [8]. Therefore, discovery of lipase inhibitors from herbal medicines may provide a potential alternative for the treatment of obesity.

Ophiopogonis Radix, also named Maidong in Chinese, is the dried root of *Ophiopogon japonicus* (L.f) Ker-Gawl. and has been used as traditional Chinese medicine for a long time. It is mainly distributed and cultivated in the Sichuan and Zhejiang provinces of China, where it is called Chuanmaidong (CMD) and Zhemaidong (ZMD), respectively. Usually, CMD is harvested one year after planting, whereas ZMD requires two to three years to be harvested. Previous reports have demonstrated that saponins, homoisoflavonoids, and polysaccharides were the main bioactive constituents of Ophiopogonis Radix, which exhibited various pharmacological activities including cardio-protection, anti-inflammation, anti-cancer, antioxidant, and anti-diabetes [9]. In recent years, it was also reported that Ophiopogonis Radix could alleviate hyperlipidemia [10,11,12]. However, the lipase inhibitory activity of Ophiopogonis Radix, as well as the associations between the components and the effect, have not been reported. Thus, it is necessary to develop a fast and efficient method to evaluate the lipase inhibitory effect and uncover the anti-lipase compounds in Ophiopogonis Radix.

Several techniques have been described for the screening of lipase inhibitors, such as fluorescence assay [13], liquid chromatography-mass spectrometry [14], capillary electrophoresis [15], and lipase immobilization [16]. However, they may suffer from false-positive results, expensive instruments and/or sophisticated procedures. Thin layer chromatography (TLC) is one of the earliest chromatographic techniques developed and it is still one of the most applied techniques in pharmaceutical analysis. It can detect most compounds with just simple techniques, provide visualized results under UV light or daylight with or without chromogenic reagents, and analyze multiple samples parallelly in a run. Moreover, TLC-bioautography provides a high-throughput, inexpensive and rapid method to fish out potential bioactive compounds from medicinal herbs directly, and have been widely employed for screening of natural products with bioactivities such as anti-microbial [17], acetylcholinesterase inhibition [18,19], glucosidase inhibition [20,21], lipase inhibition [22,23], free radical scavenging, and antioxidation [17,24].

**Figure 1:**
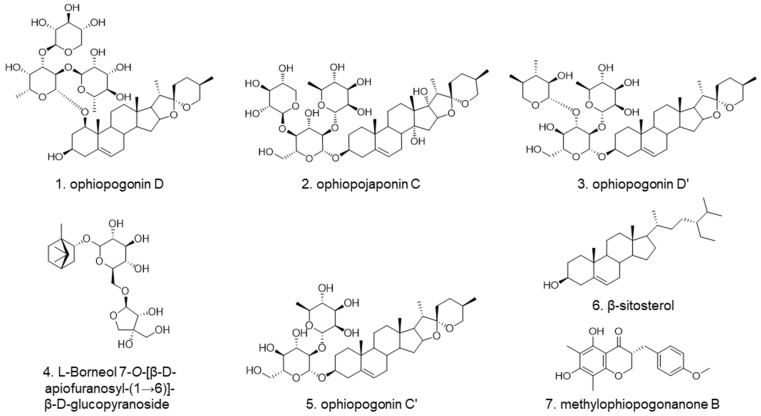
Chemical structures of seven investigated compounds.

In this study, a high-performance thin layer chromatography (HPTLC) method by two step gradient elution with two mobile phases was developed for the simultaneous separation of seven components, including ophiopogonin D (**1**), ophiopojaponin C (**2**), ophiopogonin D’ (**3**), l-borneol-7-*O*-[β-d-apiofuranosyl(1→6)]-β-d-glucopyranoside (**4**), ophiopogonin C’ (**5**), β-sitosterol (**6**), and methylophiopogonanone B (**7**) in Ophiopogonis Radix (Figure 1). Chemometric analysis was performed to discriminate the samples from different regions and/or growth years. Then HPTLC-bioautographic method was used to evaluate the lipase inhibition activities and screen potential lipase inhibitors from Ophiopogonis Radix. Furthermore, the possible binding modes and interactions between the active compounds and lipase were revealed by molecular docking analysis.

## 2. Results and Discussion

### 2.1. Optimization of the HPTLC Conditions

Mobile phase and migration distance were optimized to achieve good separation. Toluene-methanol-acetic acid (80:5:0.1, *v*/*v*/*v*) and ethyl acetate-toluene (10:90 *v*/*v*) are used as the mobile phase in Chinese Pharmacopoeia (2020 edition) [25] and European Pharmacopoeia (10th edition) [26], respectively, for the identification of Ophiopogonis Radix, which are only suitable for the low-polarity constituents such as homoisoflavonoids and β-sitosterol. While in Hong Kong Chinese Materia Medica Standards, TLC analysis is performed by using dichloromethane-methanol-water (8:2:0.3, *v*/*v*/*v*) as the mobile phase for the identification of ophiopogonin D, a polar saponin [27], but low-polarity components could not be separated under this condition. Several other mobile phases were also tried, including toluene-dichloromethane-methanol-water (11:72:17:2, *v*/*v*/*v*/*v*), cyclohexane-toluene-ethyl acetate-formic acid-acetic acid-water (2.5:5:7:2:2:1, *v*/*v*/*v*/*v*/*v*/*v*), and toluene-ethyl acetate-formic acid-water (9:11:2.9:1, *v*/*v*/*v*/*v*). However, the seven components could not be separated well in one development due to their significantly different polarity. Hence, two step gradient elution with two mobile phases was utilized to satisfy the needs of simultaneous separation of low- and high-polarity compounds. For the development of high-polarity compounds, dichloromethane-methanol-ethyl acetate-water (70:25:12:3, *v*/*v*/*v*/*v*) was chosen as the mobile phase, which was modified based on the reported methods [27,28]. For the development of low-polarity compounds, the mobile phase of dichloromethane-methanol (300:1, *v*/*v*) adjusted from the previous report [29] was used.

Different migration distances were evaluated to obtain the best resolution of the investigated components. For the first development, a series of eluting distances (50 mm, 55 mm, 60 mm, 65 mm and 70 mm) were tested, and 60 mm was chosen as the ideal one, and the total eluting distance was set as 90 mm.

**Figure 2:**
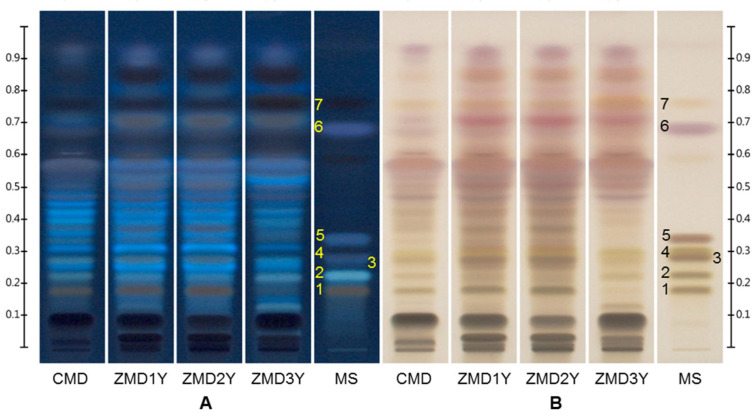
Typical HPTLC chromatograms of mixed standards and Ophiopogonis Radix samples analyzed by two step gradient elution with two mobile phases. Plates were sprayed with 10% sulfuric acid in ethanol solution and viewed under UV 366 nm (**A**) and white light (**B**). CMD: CMD (one-year-old); ZMD 1Y: one-year-old ZMD; ZMD2Y: two-year-old ZMD; ZMD3Y: three-year-old ZMD; MS: mixed standards. 1. ophiopogonin D (R_f_ 0.18), 2. ophiopojaponin C (R_f_ 0.23), 3. ophiopogonin D’ (R_f_ 0.28), 4. l-borneol-7-*O*-[β-d-apiofuranosyl(1→6)]-β-d-glucopyranoside (R_f_ 0.30), 5. ophiopogonin C’ (R_f_ 0.34), 6. β-sitosterol (R_f_ 0.69), and 7. methylophiopogonanone B (R_f_ 0.78).

As a result, the high-polarity compounds including ophiopogonin D (**1**), ophiopojaponin C (**2**), ophiopogonin D’ (**3**), l-borneol-7-*O*-[β-d-apiofuranosyl(1→6)]-β-d-glucopyranoside (**4**), and ophiopogonin C’ (**5**) were separated by the high-polarity mobile phase in the first run, while the low-polarity compounds were developed as one main band in the front. Then the low-polarity components, including β-sitosterol (**6**) and methylophiopogonanone B (**7**) were further resolved using the low-polarity mobile phase in the second run, while the high-polarity compounds could not be driven. Finally, seven investigated components in Ophiopogonis Radix were well separated by the developed HPTLC method (Figure 2 and Figure S1). Compared with the current official standards that focus on low-polarity constituents or only ophiopogonin D, the developed method could be used for the detection of low- and high- polarity components simultaneously, which provided a more comprehensive means for quality evaluation of Ophiopogonis Radix.

### 2.2. Comparison of Ophiopogonis Radix from Different Regions and/or Growth Years by Chemometric Approaches

Ophiopogonis Radix samples collected from Sichuan and Zhejiang provinces of China with different growth years were analyzed by the developed HPTLC method, and their HPTLC profiles were shown in Figure 2 and Figure S1. It was shown that the profiles of CMD and ZMD were quite different, especially for the bands in the range of R_f_ 0.68–0.94, the contents in CMD were lower than those in ZMD. On the other hand, there was no obvious difference among different CMD samples, but growth years had an important influence on the constituents of ZMD samples. The contents of ophiopogonin D, ophiopogonin D’, and bands of R_f_ 0.37 and 0.43 in one- and two- year-old ZMD samples were much higher than those in three-year-old ZMD samples, while the contents of ophiopojaponin C and bands of R_f_ 0.78 containing methylophiopogonanone B increased with growth years, and their corresponding bands in three-year-old ZMD samples became much clearer than those in younger ones.

**Figure 3:**
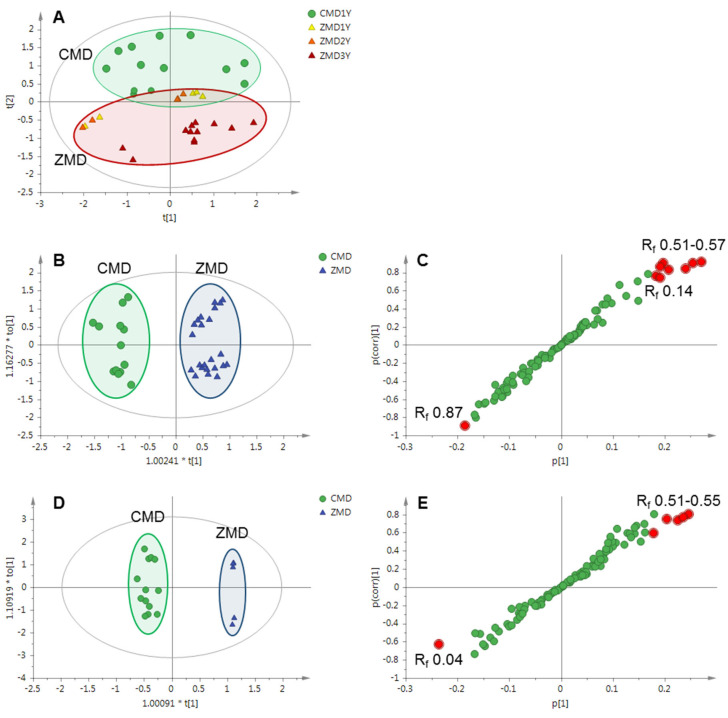
Multivariate analysis based on the HPTLC profiles of CMD and ZMD. (**A**) PCA score plot of CMD and ZMD (R^2^X = 0.902, Q^2^ = 0.716), (**B**) OPLS-DA score plot of CMD and ZMD (R^2^X = 0.621, R^2^Y = 0.945, Q^2^ = 0.906), (**C**) OPLS-DA S-plot of CMD and ZMD, (**D**) OPLS-DA score plot of one-year-old CMD and ZMD (R^2^X = 0.772, R^2^Y = 0.983, Q^2^ = 0.917), (**E**) OPLS-DA S-plot of one-year-old CMD and ZMD. The variables with VIP > 1.5 in the S-plots were highlighted with red filled circles.

HPTLC images (366 nm) were extracted with rTLC program and further analyzed by SIMCA software [30]. As shown in Figure 3A, clear separations were observed among the samples collected from Sichuan and Zhejiang provinces with principal component analysis (PCA), an unsupervised multivariate statistical method for pattern recognition. The model consisted of eight principal components explaining 90.2% of the total variance (R^2^X = 0.902), with a Q^2^ value of 0.818. Interestingly, one- and two-year-old ZMD samples were closer to CMD samples that were also of one-year-old. To further explore the differences among the samples, supervised statistical modeling methods including orthogonal partial least square-discriminant analysis (OPLS-DA) and orthogonal partial least square (OPLS) were employed. These models were characterized by high goodness-of-fit (R^2^Y values between 0.910 and 0.983) and predictive ability (Q^2^ values in the range of 0.859−0.917). Firstly, samples of Ophiopogonis Radix were divided into two classes based on the production regions (Sichuan and Zhejiang province) by OPLS-DA (Figure 3B). The S-plot indicated that the bands of R_f_ 0.51–0.57, 0.14, and 0.87 contributed a lot to the distinction of CMD and ZMD (Figure 3C). In order to exclude the influence of growth years, one-year-old CMD and one-year-old ZMD samples were classified into two groups by OPLS-DA, the results suggested that the bands of R_f_ 0.51–0.55 and 0.04 were the most different components between them (Figure 3D,E).

**Figure 4:**
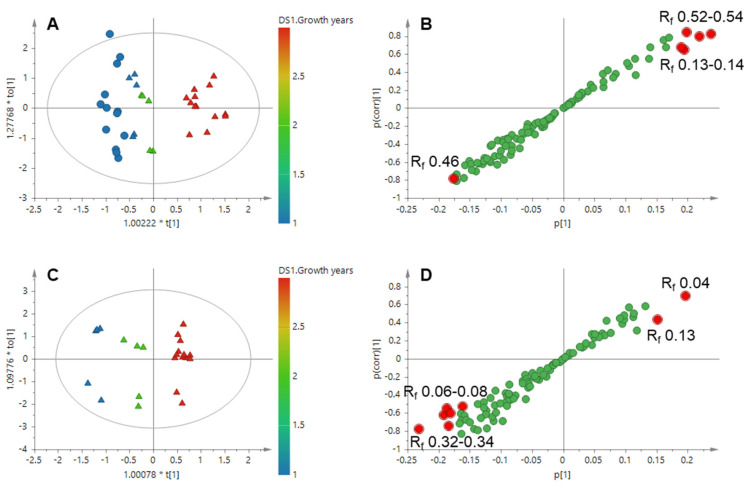
OPLS based on the HPTLC profiles of CMD and ZMD samples with different growth years. (**A**) OPLS score plot of CMD and ZMD (R^2^X = 0.601, R^2^Y = 0.910, Q^2^ = 0.859), (**B**) OPLS S-plot of CMD and ZMD, (**C**) OPLS score plot of ZMD with different growth years (R^2^X = 0.849, R^2^Y = 0.978, Q^2^ = 0.897), (**D**) OPLS S-plot of ZMD with different growth years. The variables with VIP > 1.5 in the S-plots were highlighted with red filled circles.

Then, the samples with different growth years were clustered into three groups by OPLS using the growth years as Y-variables (Figure 4A). The band of R_f_ 0.46 was found to be the characteristic component in younger samples while the bands of R_f_ 0.13–0.14 and 0.52–0.54 were higher in older samples (Figure 4B). Excluding the region factor, ZMD samples were divided into three classes based on their growth years (Figure 4C). The bands of R_f_ 0.06–0.08 and 0.32–0.34 were higher in younger ZMD samples and the bands of R_f_ 0.04 and 0.13 were strongly correlated with the older ZMD (Figure 4D). The band of R_f_ 0.34 could be identified as ophiopogonin C’ by comparison with reference standards.

There were a series of reports regarding the comparison of Ophiopogonis Radix from different regions. Generally, it was considered that CMD contained higher levels of steroidal saponins and ZMD had higher contents of homoisoflavonoids [31,32,33], but there were few reports on the samples from different growth years [28]. Our study showed that the growth year was also an important factor on the constituents of Ophiopogonis Radix. For example, the content of ophiopogonin D, a main saponin of Ophiopogonis Radix, was reported to be much higher in CMD than that in ZMD [32,33], but actually it was high in one- and two-year-old ZMD, but low in three-year-old ZMD. Therefore, the differences between CMD and ZMD were not only from their different producing areas but also from their different growth years.

### 2.3. Screening of Lipase Inhibitors from Ophiopogonis Radix by HPTLC-Bioautography

**Figure 5:**
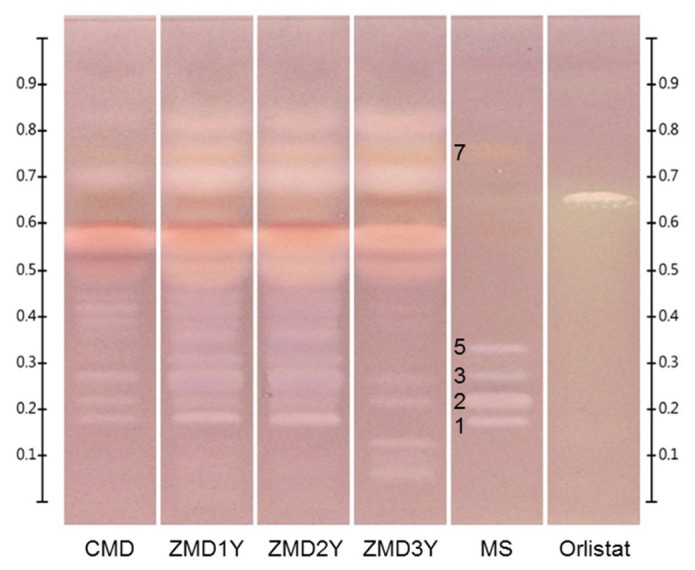
Typical HPTLC bioautograms of lipase inhibition assays. Plates were viewed under white light. CMD: one-year-old CMD, ZMD 1Y: one-year-old ZMD, ZMD2Y: two-year-old ZMD, ZMD3Y: three-year-old ZMD, MS: mixed standards. 1. ophiopogonin D (R_f_ 0.18), 2. ophiopojaponin C (R_f_ 0.23), 3. ophiopogonin D’ (R_f_ 0.28), 5. ophiopogonin C’ (R_f_ 0.34), 7. methylophiopogonanone B (R_f_ 0.78), Orlistat: positive control.

The effects of sampling volumes (6–15 μL), lipase concentration (30–120 U/mL) and Fast Blue B salt (FBB) concentration (1–2 mg/mL) on the HPTLC bioautographic assay were investigated and the optimum conditions were: sampling volumes, 6 μL; lipase concentration, 60 U/mL; and FBB concentration, 2.0 mg/mL. The obtained HPTLC bioautograms of different Ophiopogonis Radix samples were shown in Figure 5 and Figure S1. The profiles of CMD, one- and two-year-old ZMD were similar, and the inhibitory activities of the latter two were stronger due to the clearer bands after the reaction. The white bands of R_f_ 0.18–0.45 (saponins), 0.70, 0.78 (homoisoflavonoids) and 0.83 were the components with potential lipase inhibitory effect. The profiles of three-year-old ZMD samples were significantly different from those of the other samples, especially in the region of R_f_ 0.05–0.45, and the white bands of R_f_ 0.05–0.13 could be only observed in three-year-old ZMD samples. After comparison with reference standards, five of the white bands were identified as ophiopogonin D (R_f_ 0.18), ophiopojaponin C (R_f_ 0.23), ophiopogonin D’ (R_f_ 0.28), ophiopogonin C’ (R_f_ 0.34), and methylophiopogonanone B (R_f_ 0.78).

### 2.4. Molecular Docking Studies

**Figure 6:**
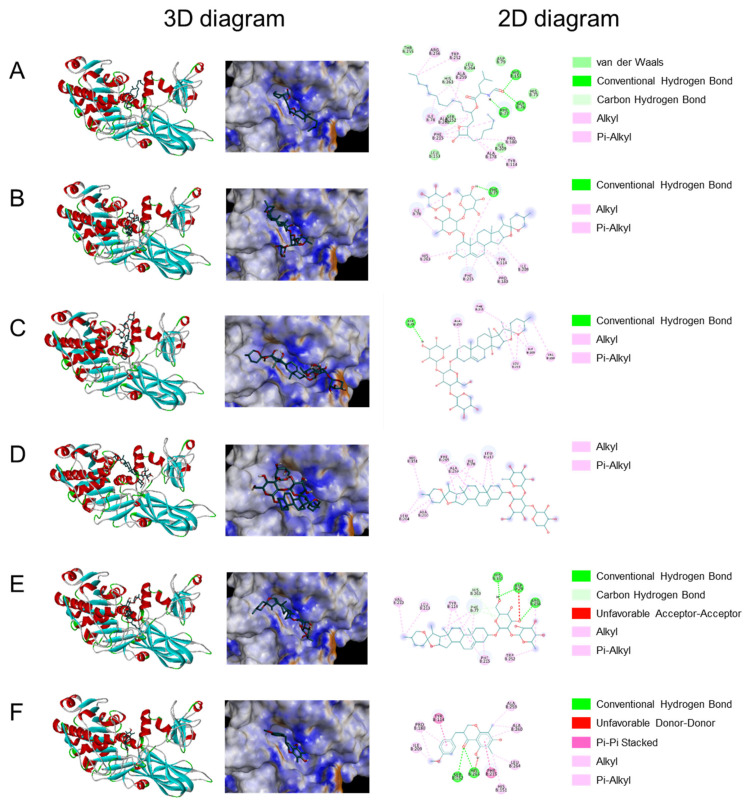
Binding modes and interactions between orlistat (**A**), ophiopogonin D (**B**), ophiopojaponin C (**C**), ophiopogonin D’ (**D**), ophiopogonin C’ (**E**), methylophiopogonanone B, (**F**) and pancreatic lipase.

Molecular docking was used to explore the possible binding sites and modes of action between lipase and the active compounds including ophiopogonin D, ophiopojaponin C, ophiopogonin D’, ophiopogonin C’, and methylophiopogonanone B. As shown in Table 1, the binding energies of the five compounds with lipase were all less than −5.0 kcal/mol, indicating their good binding affinity with lipase. Moreover, ophiopogonin D, ophiopogonin C’, and methylophiopogonanone B showed better binding affinity than orlistat, the positive drug. The 3D and 2D interaction diagrams of the compounds with lipase were shown in Figure 6. The results demonstrated that these five compounds combined with the lipase mainly through hydrogen bond, alkyl and Pi-alkyl interaction, etc. Based on the 3D diagram, ophiopojaponin C and ophiopogonin D’ could not fit the pocket well, which caused their binding affinity to be relatively low.

## 3. Materials and Methods

### 3.1. Materials and Chemicals

The Ophiopogonis Radix samples were collected from Sichuan and Zhejiang provinces of China, and the detailed information was listed in Table 2. The botanical origin of materials was identified by Dr. Xiao-Jia Chen, one of the authors. All voucher specimens were deposited at the Institute of Chinese Medical Sciences, University of Macau, Macao SAR, China.

All chemicals and solvents were of analytical grade. Dichloromethane and sulfuric acid (98%) were purchased from Merck (Darmstadt, Germany). Methanol and ethanol were purchased from Damao Chemical Reagent Factory (Tianjin, China). Ethyl acetate was obtained from ACI Lascan Limited (Bangkok, Thailand). Petroleum ether (60~90 °C) was obtained from Xilong Scientific Co., Ltd. (Shantou, China). Ophiopogonin D’ (HPL ≥ 98%) and methylophiopogonanone B (HPLC ≥ 98%) were purchased from Baoji Herbest Biotech Co., Ltd. (Baoji, China). Ophiopogonin D (HPLC ≥ 98%) were obtained from Chengdu Pufei De Biotech Co., Ltd. (Chengdu, China), and β-sitosterol (HPLC ≥ 98.80%) was obtained from Chengdu Must Bio-technology Co., Ltd. (Chengdu, China). Ophiopogonin C’ (HPLC ≥ 98%) and Fast Blue B salt (dry content ≥ 95%) were purchased from Shanghai Yuanye Bio-Technology Co., Ltd. (Shanghai, China), and ophiopojaponin C (HPLC ≥ 98%) was purchased from Chengdu DeSiTe Biological Technology Co., Ltd. (Chengdu, China). Tris was offered by Bio-Rad Laboratories (Hercules, CA, USA). β-Naphthyl myristate (>97.0%) and orlistat (>97.0%) were offered by TCI (Shanghai) Development Co., Ltd. (Shanghai, China). Bovine serum albumin (pH 7, ≥98.0%) and lipase (from porcine pancreas) were purchased from Sigma Aldrich (St. Louis, MO, USA). All aqueous solutions were prepared with deionized water purified by Millipore Milli Q-Plus system (Millipore, Billerica, MA, USA).

### 3.2. Preparation of Sample and Standard Solutions

Dried powdered samples (4.0 g) were sonicated with 40 mL of anhydrous ethanol for 30 min, then the supernatants were evaporated to dryness in a rotary evaporator after centrifugation at 4000 rpm for 15 min. The residue was dissolved in 1.0 mL anhydrous ethanol, and the collected solution filtered through a 0.22 μm membrane was used for the analysis.

Mixed standards solution was prepared by dissolving ophiopogonin C’, ophiopojaponin C, ophiopogonin D, ophiopogonin D’, l-borneol-7-*O*-[β-d-apiofuranosyl(1→6)]-β-d-glucopyranoside, β-sitosterol and methylophiopogonanone B in methanol at the concentration of 0.5 mg/mL.

### 3.3. HPTLC Analysis

All analyses were conducted by a CAMAG TLC system (CAMAG, Switzerland) containing an automatic TLC sampler 4 with a 25 μL syringe, a TLC visualizer equipped with visionCATS (version 2.5) software, a chromatogram immersion device III, and a TLC plate heater III. The HPTLC separations were performed on 20 × 10 cm HPTLC silica gel 60 F_254_ plates (Merck, Darmstadt, Germany). The samples and mixed standards solution (4 μL) were applied as 6 mm bands and 10 mm from the bottom edge on the HPTLC plates. The syringe was washed with methanol three times between the applications of each substance. Firstly, the applied HPTLC plate was pre-saturated with dichloromethane-methanol-ethyl acetate-water (70:25:12:3, *v*/*v*/*v*/*v*) for 25 min in a glass twin trough chamber and developed over a path of 60 mm for the separation of high-polarity components. After the plate was dried under an airstream, the same plate was pre-equilibrated with dichloromethane-methanol (300:1, *v*/*v*) for 15 min in a twin trough chamber and developed to the distance of 90 mm to isolate the low-polarity compounds. Then, the developed plate was dried in a stream of cool air, immersed in 10% sulfuric acid in ethanol solution for one second, heated for 10 min at 105 °C on a TLC plate heater, and subsequently recorded under UV (366 nm) and white light.

### 3.4. Chemometric Analysis

The obtained HPTLC images were extracted using the rTLC V.1.0 program (http://shinyapps.ernaehrung.uni-giessen.de/rtlc/, accessed on 24 December 2021) for acquiring data matrix [30]. The parameters were adjusted to extract every track on the basis of sample application. And data of the gray channel was further analyzed by multivariate data analysis using SIMCA software (version 14.1, Umetrics).

### 3.5. HPTLC-Bioautographic Assay

Lipase (60 U/mL) was prepared in 100 mL of 50 mM Tris-HCl buffer (pH 7.4) with 100 mg bovine serum albumin added to stabilize the enzyme during the bioassay. The lipase solution was freshly prepared before use and kept at 4 °C in darkness. β-Naphthyl myristate (4 mM) dissolved in petroleum ether (60~90 °C) was used as the substrate. Orlistat (2.5 mg/mL) prepared in methanol was used as the positive control for detecting lipase inhibitory activity. FBB was prepared in deionized water as the chromogenic agent.

The plate was developed by the HPTLC method as mentioned above and dried under a stream of cool air until complete removal of the solvent. Then the plate was dipped in β-naphthyl myristate solution, followed by being air-dried to remove petroleum ether. After being sprayed with 60 U/mL lipase solution, the plate was then incubated for 20 min at 37 °C in a closed incubation chamber containing a small amount of water to keep high humidity but without direct contact with water. Finally, the plate was sprayed with 2 mg/mL FBB aqueous solution to give a purple coloration. The plate was dried with cool air until no free liquid was on it, the bands with lipase inhibitory activity were observed as white yellow or brown bands on a purple background and recorded under white light.

### 3.6. Molecular Docking Studies

Virtual molecular docking was employed to analyze the potential binding modes of lipase and the active compounds. The crystal structure of pancreatic lipase (PDB: 1LPB) was downloaded from the RCSB PDB (https://www.rcsb.org/, accessed on 24 December 2021) [34] with 2.46Å, which was selected and saved as *pdb* format. The ligand and receptor were split by Pymol. Autodock Tools 1.5.6 was used to transform *pdb* to *pdbqt* format files with gird boxes adjusted to cover the entire pocket for the preparation of virtual docking. The Structures of chemicals were collected from Pubchem [35] as *sdf* format and transformed into *pdbqt* format. Autodock Vina1.1.2 [36] was used to simulate the potential interactions among the selected compounds and the targets.

## 4. Conclusions

In this study, an HPTLC method by two step gradient elution combined with multivariate statistical analysis was developed to analyze and compare different Ophiopogonis Radix samples, which indicated that both regions and growth years could greatly influence the components of the herb. Furthermore, the potential lipase inhibitors were screened by HPTLC-bioautography and validated via molecular docking analysis. The developed HPTLC method provided an alternative tool for quality control of Ophiopogonis Radix and screening of the potential lipase inhibitors. The five compounds with lipase inhibitory activity could be used as the quality markers of Ophiopogonis Radix or as bioactive components for the development of related hypolipidemic products.

## Figures and Tables

**Table 1:** Molecular docking results of lipase and the identified active compounds.

Compounds	Binding Energy (kcal/mol)	Hydrogen Bonds	Other Amino Acid Residues
Orlistat	−7.2	HIS151, GLY76, PHE77	THR255, ARG256, TRP252, HIS263, ALA259, LEU264, ASP79, HIS75, ILE78, ALA260, SER152, PHE215, LEU153, ALA178, TYR114, ILE209, PRO180
Ophiopogonin D	−9.6	PHE77	ILE78, HIS263, PHE215, PRO180, TYR114, ILE209
Ophiopojaponin C	−6.7	ASP79	ALA259, PHE215, LEU213, ILE209, VAL210
Ophiopogonin D’	−6.5	-	HIS151, PHE215, ALA259, ILE78, LEU213, LEU264, ALA260
Ophiopogonin C’	−8.4	HIS151, ASP79, ARG256	VAL210, LEU213, TYR114, HIS263, PHE77, PHE215, TRP252
Methylophiopogonanone B	−9.4	SER152, HIS263	ILE209, PRO180, TYR114, ALA259, ALA260, PHE215, LEU264, HIS151

**Table 2:** Information of Ophiopogonis Radix samples.

No.	Code	Regions	Growth Years
1	C1	Laoma, Santai, Sichuan, China	1
2	C2	Laoma, Santai, Sichuan, China	1
3	C3	Laoma, Santai, Sichuan, China	1
4	C4	Laoma, Santai, Sichuan, China	1
5	C5	Laoma, Santai, Sichuan, China	1
6	C6	Laoma, Santai, Sichuan, China	1
7	C7	Xinde, Santai, Sichuan, China	1
8	C9	Huanyuan, Santai, Sichuan, China	1
9	C10	Laoma, Santai, Sichuan, China	1
10	C11	Xinde, Santai, Sichuan, China	1
11	C12	Laoma, Santai, Sichuan, China	1
12	C13	Guangming, Santai, Sichuan, China	1
13	C14	Lingxing, Santai, Sichuan, China	1
14	ZA1	Muliwan, Sanmen, Zhejiang, China	1
15	ZA2	Muliwan, Sanmen, Zhejiang, China	2
16	ZA3	Muliwan, Sanmen, Zhejiang, China	3
17	ZB1	Kanxiajin, Sanmen, Zhejiang, China	1
18	ZB2	Kanxiajin, Sanmen, Zhejiang, China	2
19	ZB3	Kanxiajin, Sanmen, Zhejiang, China	3
20	ZC1	Baixi, Sanmen, Zhejiang, China	1
21	ZC2	Baixi, Sanmen, Zhejiang, China	2
22	ZC3	Baixi, Sanmen, Zhejiang, China	3
23	ZD1	Nanlin, Sanmen, Zhejiang, China	1
24	ZD2	Nanlin, Sanmen, Zhejiang, China	2
25	ZD3	Nanlin, Sanmen, Zhejiang, China	3
26	ZE1	Xiyang, Sanmen, Zhejiang, China	1
27	ZE2	Xiyang, Sanmen, Zhejiang, China	2
28	ZE3	Xiyang, Sanmen, Zhejiang, China	3
29	ZF1	Fuhai, Cixi, Zhejiang, China	3
30	ZF2	Fuhai, Cixi, Zhejiang, China	3
31	ZF3	Fuhai, Cixi, Zhejiang, China	3
32	ZG1	Shengshan, Cixi, Zhejiang, China	3
33	ZG2	Shengshan, Cixi, Zhejiang, China	3
34	ZG3	Shengshan, Cixi, Zhejiang, China	3
35	ZG4	Shengshan, Cixi, Zhejiang, China	3
36	ZG5	Shengshan, Cixi, Zhejiang, China	3

## Data Availability

The data presented in this study are contained within the article.

## Supplementary Materials

The following supporting information can be downloaded online, Figure S1: HPTLC chromatograms (**A**,**B**) and HPTLC bioautograms (**C**) of mixed standards and Ophiopogonis Radix samples. Sample codes are the same as in Table 2. MS: mixed standards. 1. ophiopogonin D, 2. ophiopojaponin C, 3. ophiopogonin D’, 4. L-borneol-7-*O*-[β-D-apiofuranosyl(1→6)]-β-D-glucopyranoside, 5. ophiopogonin C’, 6. β-sitosterol, and 7. methylophiopogonanone, Orlistat: positive control.

## Sample Availability

Samples of the compounds are available from the authors.
