# Supplementary material for: Rapid Screening of Lipase Inhibitors from Ophiopogonis Radix Using High-Performance Thin Layer Chromatography by Two Step Gradient Elution Combined with Bioautographic Method
Source: Molecules. 2022 Feb 9;27(4):1155. doi: 10.3390/molecules27041155 (PMC8878968; doi:10.3390/molecules27041155)
Supplement: Supplementary file 1 [file molecules-27-01155-s001.zip › molecules-1551076-supplementary.pdf]

Rapid Screening of Lipase Inhibitors from Ophiopogonis Radix Using High-Performance Thin Layer Chromatography by Two Step Gradient Elution Combined with Bioautographic Method

Xue Hua [1,†], Hui-Jie Hong [1,†], Dai-Yan Zhang [1], Qiao Liu [1], Fong Leong [1], Qi Yang [1], Yuan-Jia Hu [1,*] and Xiao-Jia Chen [1,2,*]

[1] Institute of Chinese Medical Sciences, and State Key Laboratory of Quality Research in Chinese Medicine, University of Macau, Macao SAR, China; mb75819@connect.um.edu.mo (X.H.); yc17534@ um.edu.mo (H.-J.H.); yc07528@um.edu.mo (D.-Y.Z.); yb87502@um.edu.mo (Q.L.); yb37501@um.edu.mo (F.L.); mc05849@um.edu.mo (Q.Y.)

[2] Zhuhai UM Science & Technology Research Institute, Zhuhai 519031, China

* Correspondence: YuanjiaHu@um.edu.mo (Y.-J.H.); XiaojiaChen@um.edu.mo (X.-J.C.); Tel.: +853-88228507 (Y.-J.H.); +853-88224915 (X.-J.C.)

† These authors contributed equally to this work.

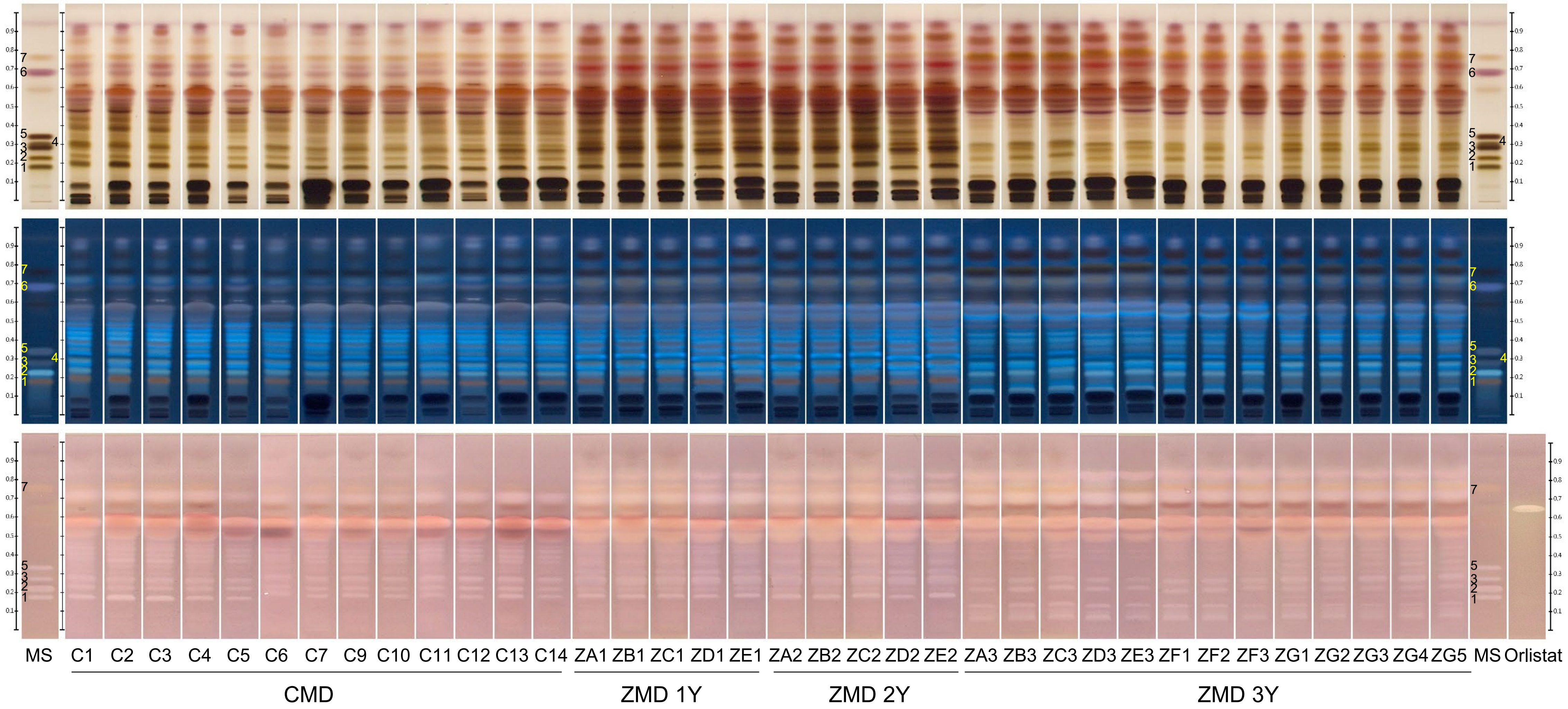

Figure S1: HPTLC chromatograms (**A** and **B**) and HPTLC bioautograms (**C**) of mixed standards and Ophiopogonis Radix samples. Sample codes are the same as in Table 2. MS: mixed standards. 1. ophiopogonin D, 2. ophiopojaponin C, 3. ophiopogonin D', 4. L-borneol-7-*O*-[β-D-apiofuranosyl(1→6)]-β-D-glucopyranoside, 5. ophiopogonin C', 6. β-sitosterol, and 7. methylophiopogonanone, Orlistat: positive control.
